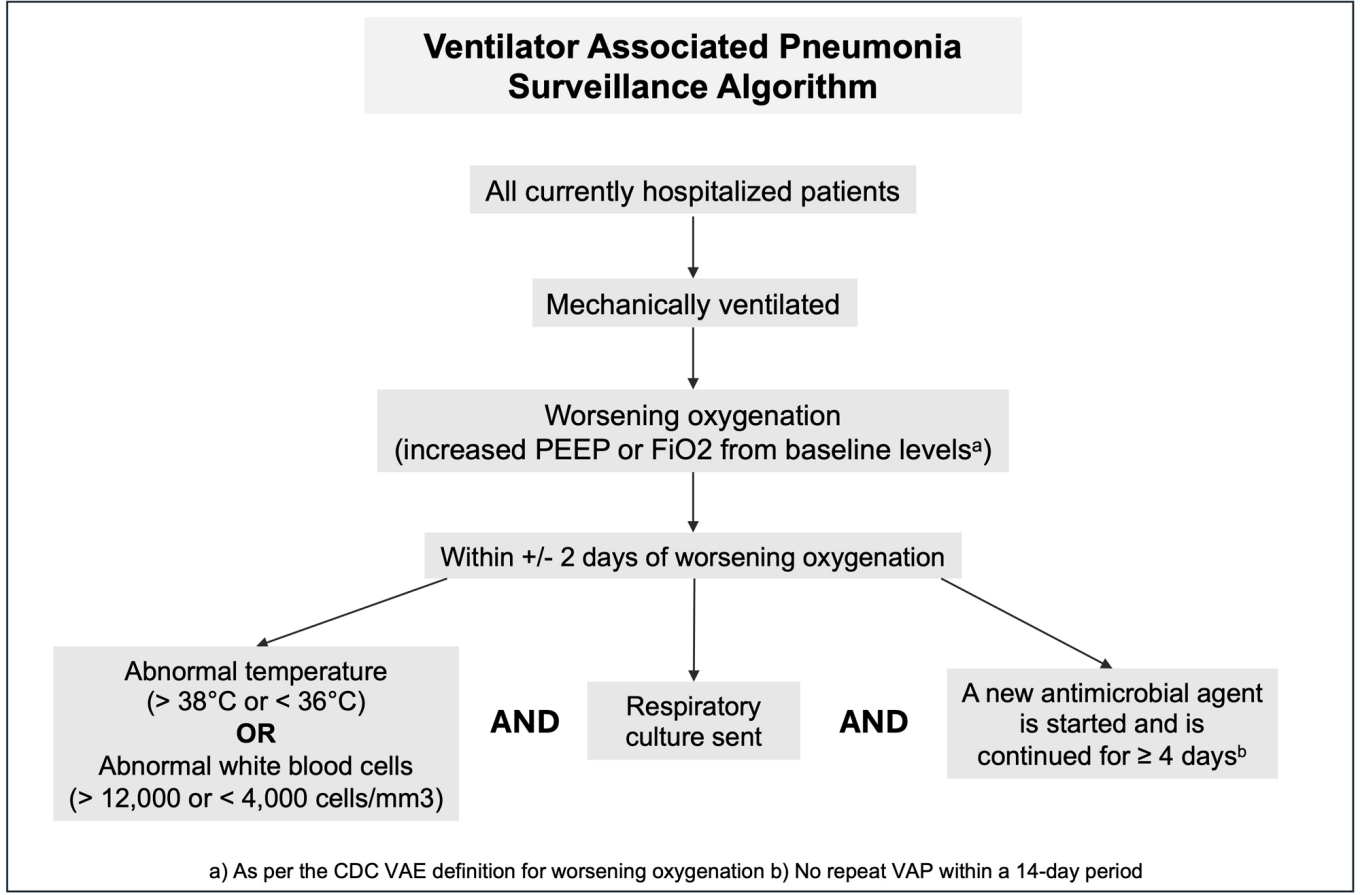# An Improved Algorithm for the Detection of Ventilator Associated Pneumonia

**DOI:** 10.1017/ash.2024.330

**Published:** 2024-09-16

**Authors:** Dan Ding, Heidy Wang, Madeline DiLorenzo, Sarah Hochman, Corinne Thompson, Michael Phillips, Sherif Shoucri

**Affiliations:** NYU Langone Health; NYU Grossman School of Medicine

## Abstract

**Background:** Ventilator associated pneumonia (VAP) is associated with significant rates of morbidity and all-cause mortality. Active VAP surveillance can identify risk factors for which targeted preventive measures can be implemented. However, surveillance efforts are complicated by challenges associated with accurate VAP diagnosis. We aimed to improve the accuracy and automation of existing VAP diagnostic algorithms to better identify patients at risk. **Methods:** The study was conducted at NYU Langone Health from June 2022 through December 2023. We created a semi-automated VAP surveillance system using the Centers for Disease Control & Prevention (CDC) ventilator associated event (VAE) definition as a base framework (Figure 1). We modified this definition to include additional elements, such as having a sputum culture ordered within 48 hours of worsening oxygen status, regardless of culture result. Using this algorithm—followed by manual clinician reviews—we retrospectively assessed possible VAP cases to determine the ability of our surveillance system to correctly identify VAP. **Results:** Of the 123 possible VAP cases identified through our automated system, 75 (61%) were correctly diagnosed as VAP after clinical review. This reflects a rate of 1.5 infections per 1000 ventilation days across the system and 1.85 infections per 100 patients ventilated for greater than 2 days. Of the 48 remaining patients without VAP after clinical review, 25% (n=12) were characterized as having hospital-acquired pneumonia, 21% (n=10) as acute respiratory distress syndrome or infection at another site and 10% (n=5) as pulmonary embolism/infarction. Among all patients identified through this automated system (VAP and non-VAP), 53% experienced in-hospital death. **Discussion:** Our automated VAP surveillance algorithm identified 123 cases of potential VAP, 61% of which were consistent with a clinical diagnosis of VAP upon manual chart review. Our VAP rate of 1.5 infections per 1000 ventilation days was similar to published rates at other North American hospital systems. The high in-hospital mortality rate among these patients highlights the need for improved surveillance systems and earlier interventions to reduce the risk of VAP. There are several limitations to the CDC’s VAE definition, including its requirement of a positive microbiologic culture and focus on sputum quality. This potentially misses cases of culture-negative VAP in patients receiving antibiotics prior to sputum collection. Our goal is to continue to validate and improve our algorithm’s ability to correctly identify patients with clinical VAP, so that targeted prevention efforts can be focused upon the patients with the highest risk for poor outcomes.

**Disclosure:** Madeline DiLorenzo: Stocks - Abbvie, Amgen Inc., Becton Dickinson, Biogen Inc., Bristol Myers and Squibb, CVS Health, Davita Inc., Elevance Health, Gilead, Henry Schein, Hologic Inc., Humana Inc., Jazz Pharmaceuticals, Laboratory Corp, Merck and Co., Quest Diagnostics, ResMed Inc., Teladoc Health, Vertex Pharmaceuticals, West Pharmaceuticals

**Figure:**